# The Roles of Cyclin-Dependent Kinases in Cell-Cycle Progression and Therapeutic Strategies in Human Breast Cancer

**DOI:** 10.3390/ijms21061960

**Published:** 2020-03-13

**Authors:** Lei Ding, Jiaqi Cao, Wen Lin, Hongjian Chen, Xianhui Xiong, Hongshun Ao, Min Yu, Jie Lin, Qinghua Cui

**Affiliations:** 1 Lab of Biochemistry & Molecular Biology, School of Life Sciences, Yunnan University, Kunming 650091, China; dingleiynu@ynu.edu.cn (L.D.); caojiaqi027@163.com (J.C.); linwen967@163.com (W.L.); chenhj098@163.com (H.C.); xiongxianhui1995@163.com (X.X.); aohongshun@163.com (H.A.); yumin@ynu.edu.cn (M.Y.); linjie@ynu.edu.cn (J.L.); 2 Key Lab of Molecular Cancer Biology, Yunnan Education Department, Kunming 650091, China

**Keywords:** breast cancer (BC), cyclin-dependent kinase (CDK), CDK inhibitor, cell cycle, clinic therapy

## Abstract

Cyclin-dependent kinases (CDKs) are serine/threonine kinases whose catalytic activities are regulated by interactions with cyclins and CDK inhibitors (CKIs). CDKs are key regulatory enzymes involved in cell proliferation through regulating cell-cycle checkpoints and transcriptional events in response to extracellular and intracellular signals. Not surprisingly, the dysregulation of CDKs is a hallmark of cancers, and inhibition of specific members is considered an attractive target in cancer therapy. In breast cancer (BC), dual CDK4/6 inhibitors, palbociclib, ribociclib, and abemaciclib, combined with other agents, were approved by the Food and Drug Administration (FDA) recently for the treatment of hormone receptor positive (HR+) advanced or metastatic breast cancer (A/MBC), as well as other sub-types of breast cancer. Furthermore, ongoing studies identified more selective CDK inhibitors as promising clinical targets. In this review, we focus on the roles of CDKs in driving cell-cycle progression, cell-cycle checkpoints, and transcriptional regulation, a highlight of dysregulated CDK activation in BC. We also discuss the most relevant CDK inhibitors currently in clinical BC trials, with special emphasis on CDK4/6 inhibitors used for the treatment of estrogen receptor-positive (ER+)/human epidermal growth factor 2-negative (HER2−) M/ABC patients, as well as more emerging precise therapeutic strategies, such as combination therapies and microRNA (miRNA) therapy.

## 1. Introduction

Breast cancer (BC) is one of the leading causes of mortality in cancer patients, classified as five major sub-types based on the comprehensive intrinsic gene expression profiling: luminal A (estrogen receptor (ER)- and/or progesterone receptor (PR)-positive, and human epidermal growth factor receptor 2 (HER2)-negative), luminal B (ER- or PR-positive and HER2-positive), basal-like (ER-, PR-, and HER2-negative, cytokeratin 5/6-positive, and/or epidermal growth factor receptor (EGFR)-positive, 75% of triple-negative breast cancer (TNBC) (ER-, PR-, and HER2-negative) share the basal marker expression), HER2-type (ER-negative, PR-negative, and HER2-positive), and normal-like [1]. These sub-types of BC were characterized based on specific morphological patterns, biological properties, different clinical stages, and prognosis. About 77% of BC patients are receptor-positive and the targeted treatment has proven efficacy [2,3]. Unfortunately, approximately 15%–25% of TNBC patients present poor outcome due to unavailable targeted treatment. Surgery combined with chemotherapy and radiotherapy is commonly recommended for TNBC and most BC patients [4]. Early diagnosis, precise treatment, and prognosis are urgently needed to improve prognostication, prevent cancer progression, and develop efficient therapies. Currently, the role of cell-cycle regulation is a fascinating area of such discovery.

The mammalian cell division and death are the two major predominant physiologic processes in the tissue homeostasis. The cell-cycle process is highly conserved and precisely controlled to govern the genome duplication and cell cycle, consisting of four distinct ordered phases, termed G0/G1 (gap 1), S (DNA synthesis), G2 (gap 2), and M (mitosis), and multiple checkpoints to ensure faithful replication in the S phase and the exact aggregation of the chromosomes into daughter cells [5]. The G1 and G2 phases are critical regulatory checkpoints, whereby the restriction point between the G1 and S phase determines whether the cells enter the S phase or exit the cell cycle to halt at the G0 phase. The cell cycle is regulated by many cyclins and cyclin-dependent kinases (CDKs) that are a group of serine/threonine kinases. They form complexes with cyclins to stabilize, activate, and phosphorylate CDKs in the specific phases [6,7]. The formation of cyclin/CDKs controls the cell-cycle progression via phosphorylation of the target genes, such as tumor suppressor protein retinoblastoma (Rb). The activation of cyclins/CDKs is induced by mitogenic signals and inhibited by the activation of cell-cycle checkpoints in response to DNA damage [8]. The cyclin/CDKs themselves are negatively regulated by cyclin-dependent kinase inhibitors (CKIs), such as the inhibitor of CDK4 (INK4) proteins (p16^INK4a^, p15^INK4b^, p18^INK4c^, and p19^INK4d^), and CDK-interacting protein/kinase inhibitory proteins (CIP/KIPs) (p21^CIP1^, p27^KIP1^, and p57^Kip2^) [9]. In addition, the E3 ubiquitin ligases are also involved in regulating expression of many mitotic proteins to affect the transitions of the cell cycle, such as Skp1–Cul1–F-box-protein (SCF) complex and anaphase-promoting complex/cyclosome (APC/C) [10,11,12]. Dysregulation of the cell cycle and genetic alterations in cell-cycle regulatory proteins lead to uncontrolled cell proliferation in many solid cancer types, including BC [13,14], which is a frontier in biomedical research for designing synthetic inhibitors of CDKs as anticancer drugs [15]. In this review, we focus on the roles of CDKs in cell-cycle regulation and gene transcription, and we provide CDK inhibitors as potential targets in BC clinical application.

## 2. CDKs in the Cell Cycle and Transcription

CDKs respond to the extracellular and intracellular signals to regulate cell division, acting as the catalytic subunits by forming a heterodimer complex with the cyclins, which function as the regulatory subunits [16]. In human cells, there are 20 CDKs and 29 cyclins [17]. CDK1, CDK2, CDK3, CDK4, CDK6, and CDK7 directly regulate cell-cycle transitions and cell division, whereas CDK7–11 mediate gene transcription. The expression of CDKs fluctuates in a cyclical fashion throughout the cell cycle [18].

### 2.1. The Roles of CDKs in the Cell Cycle

**Figure 1:**
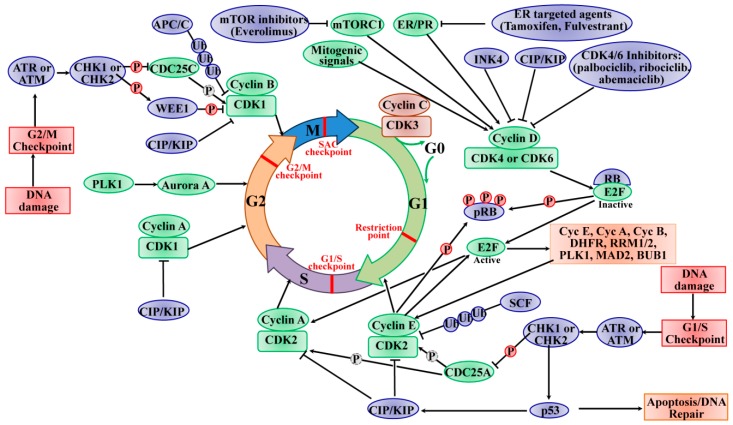
Progression of the cell cycle and its regulation by the CDKs and checkpoints. The cell cycle is regulated by many CDKs which form complexes with their associated cyclin partners. The cell cycle consists of four distinct ordered phases of the cell cycle, termed G0/G1, S, G2, and M phases, and it contains multiple checkpoints (red) throughout to prevent genomic instability, as well as ensure faithful replication. The cells exit the cell cycle and enter the reversible or permanent quiescent state (G0 phase) regulated by cyclin C/CDK3. Various extracellular signals, such as the mitogenic signal, lead to the synthesis of cyclin D and stimulate CDK4/6, resulting in promoting entry into the cell cycle. Active CDK4/6 complexes initiate the phosphorylation (P) of RB protein, thereby unleashing E2F transcription factors, resulting in the expression of cyclin E, cyclin A, cyclin B, and many genes required for S phase progression. Cyclin E subsequently activates CDK2 and contributes to the further phosphorylating RB, progresses into S phase, and initiates DNA synthesis. Near the end of S phase, cyclin A removes cyclin E and forms a new complex, cyclin A/CDK2. Cyclin A/CDK2 terminates the S phase by phosphorylating CDC6 and E2F1; it drives the cell-cycle transition from S phase to G2 phase, and subsequently activates CDK1 by cyclin A, leading to cells entering the M phase. Upon mitosis, the CDK1 activity is maintained by the complex cyclin B/CDK1. The deregulation of CDK1 enables chromosome separation and the completion of mitosis and cytokinesis. The INK4, CIP/KIP, and CDK4/6 inhibitors (palbociclib, ribociclib, and abemaciclib) inhibit the activity of CDK/cyclin. The ubiquitination (Ub) of cyclins is involved in regulating the expression of many proteins to control the cyclical activities of the CDKs, such as SCF and APC/C. The PLK1 and aurora A proteins are involved in the progression through S phase and from G2 phase into M phase. In addition, DNA damage checkpoints safeguard the genomic integrity and trigger cell-cycle arrest via checkpoint kinase 2 (CHK2) and p53 in G1 phase or via CHK1 in S or G2 phase. P in a dashed circle shows dephosphorylation. Green ovals indicate positive regulators and blue ovals indicate negative regulators of cell-cycle progression. (Adapted from reference “[8], doi:10.1038/nrc.2016.138” with permission of the journal *Nature Reviews Cancer* 2017).

In most adult tissues, the majority of cells with diploid DNA content are arrested in a quiescent G0 state that can be either transient (quiescence) or permanent (terminal differentiation or senescence). As shown in Figure 1, upon mitosis, quiescent cells are involved in re-entering the cell cycle through stimulation with mitogenic factors (hormone or growth factor). These factors converge on the cell cycle to activate cascades of intracellular signaling networks that impinge on CDK4 and CDK6 to drive cell-cycle progression from G0 or G1 phase into S phase. The activity of CDK4/6 is positively controlled by the association with D-type cyclins (cyclin D1, cyclin D2, and cyclin D3) and negatively controlled by binding to CDK inhibitors of the INK4 family (p16^INK4A^, p15^INKB^, p18^INK4C^, and p19^INK4D^) [19]. Then, the active cyclin D/CDK4/6 complexes initiate phosphorylation of tumor suppressor protein RB (encoded by RB1), as well as the closely related proteins p107 (also known as RBL1) and p130 (also known as RBL2). The RB protein originally recruits co-repressors and represses the transcription of target genes regulated by E2F transcription factors (E2Fs) to inhibit G1/S transition. In this way, the sequential phosphorylation inactivating the activity of RB leads to cell-cycle progression from G1 into S phase. Meanwhile, the phosphorylated RB de-represses E2F transcription factors and induces the transcription of G1/S target genes such as cyclin E (CCNE), CCNA, and CCNB, dihydrofolate reductase (DHFR), ribonucleotide reductase M1 (RRM1) and RRM2 and polo-like kinase 1 (PLK1), spindle checkpoint protein MAD2 (MAD2), and BUB1 mitotic checkpoint serine/threonine kinase (BUB1), involved in G1–S entry and cell-cycle progression [9]. During late G1 phase, the target genes (cyclins E1 and E2) of E2Fs are activated, thereby binding and activating CDK2, which is originally sequestered by two CDK inhibitors p21^CIP1^ and p27^KIP1^, as well as ubiquitin-mediated proteolysis of p27^KIP1^ and p21^CIP1^. In addition, cell division cycle 25A (CDC25A) activates CDK2 by removing phosphorylation from CDK2 [20]. Furthermore, the active CDK2 is capable of phosphorylating a considerably broader range of substrate profile proteins required for cell-cycle progression (such as p27^KIP1^, RB, and E2F1), DNA replication (such as replication factors A and C), centrosome duplication (such as nucleophosmin (NPM)) and histone synthesis (such as nuclear protein, coactivator of histone transcription (NPAT)) [21,22,23]. Specifically, the cyclin E/CDK2 active complex modulates RB to override the restriction point of the G1/S phase at the boundary, resulting in S phase initiation, which forms a positive feedback loop. The activities of CDK4/6 and CDK2 coordinate the cell-cycle progression into S phase, termed the “restriction point”, such that the mitogens are no longer required to complete the current cell cycle. Near the end of S phase, cyclin A removes cyclin E and forms a new complex, cyclin A/CDK2, where the cyclin E is rapidly degraded by the F-box/WD repeat-containing protein 7 (FBXW7)-mediated ubiquitylation [21,22]. The cyclin A/CDK2 complex terminates the S phase by phosphorylating CDC6 and E2F1, and drives the cell-cycle transition from S phase to G2 phase, and subsequently activates CDK1 by cyclin A leading the cells to enter the transition to M phase. Upon mitosis, CDK1 activity is maintained by the complex cyclin B/CDK1. The phosphorylation of activated CDK1 leads to the breakdown of nuclear envelope, the condensation of chromosome, and the assembly of mitotic spindle. The mitotic metaphase to anaphase is controlled by the spindle assembly checkpoints (SAC), and the anaphase is dependent on the decreased activity of CDK1 via the degradation of cyclin B by APC/C [24]. The deregulated expression of CDK1 enables chromosome separation and completion of mitosis and cytokinesis. CDK1 is the only CDK that is essential for cell-cycle progression, as it initiates the onset of mitosis and ensures the critical events occur in the exact sequence in cellular replication with high fidelity [25]. In addition to regulation by its cyclin partners, the activity of CDK1 is controlled by the balance between the WEE1 G2 checkpoint kinase (WEE1), the membrane-associated tyrosine- and threonine-specific cdc2-inhibitory kinase myelin transcription factor 1 (MYT1, also known as PKMYT1), and the phosphorylation of CDC25C phosphatases. WEE1 phosphorylates CDK1 at Tyr 15, while MYT1 is phosphorylated at Thr 14 and Tyr 15 to inhibit the activity of CDK1, and this phosphorylation is relieved by CDC25C phosphatases [26]. On the other hand, cyclin C/CDK3 phosphorylates RB to push the cells into S phase from the G0 phase. The cells exit the cell cycle and enter the reversible or permanent G0 phase, also regulated by cyclin C/CDK3 [27].

The cell cycle is arrested or blocked by DNA damage-mediated cell-cycle checkpoints, thereby allowing DNA repair before cell-cycle progression into mitosis. As shown in Figure 1, two major cell-cycle checkpoints respond to DNA damage; they occur pre- and post-DNA synthesis in G1 and G2 phases and impinge on the activity of specific CDK complexes. The checkpoint kinases phosphatidylinositol 3-kinase (PI3K)-like protein kinases (PI3KKs) ataxia telangiectasia and Rad3-related (ATR) or ataxia telangiectasia mutated (ATM) protein, and the transducer checkpoint kinases CHK1 (encoded by the CHEK1 gene) and CHK2 (encoded by the CHEK2 gene) are key regulators of DNA damage signaling [28]. The DNA damage signaling is detected by ATM/ATR, which then phosphorylate and activate CHK2/CHK1, respectively [29]. The activated CHK2 is involved in the activation of p53, leading to p53-dependent early phase G1 arrest to allow time for DNA repair [30]. The activation of p53 induces the expression of the CKI p21^CIP1^ gene, leading to inhibition of cyclin E/CDK2 complexes and downstream upregulation of DNA repair machinery. If the DNA repair cannot be completed successfully or the cells cannot program to respond to the stresses of viable cell-cycle arrest, the cells face the fate of apoptosis induced by p53 [31]. The activated CHK1 mediates temporary S phase arrest through phosphorylation to inactivate CDC25A, causing ubiquitination and proteolysis. Moreover, the activated CHK1 phosphorylates and inactivates CDC25C, leading to cell-cycle arrest in the G2 phase. The active CHK1 also directly stimulates the phosphorylation of WEE1, resulting in enhancing the inhibitory Tyr15 phosphorylation of CDK2 and CDK1 and subsequent cell-cycle blocking in G2 phase [8]. The activity of WEE1 can also be stimulated by the low levels of CDK activity in G2 cell-cycle phase [32]. The SAC, also known as the mitotic checkpoint, functions as the monitor of the correct attachment of the chromosomes to the mitotic spindle in metaphase, which is regulated by the TTK protein kinase (TTK, also known as monopolar spindle 1 (MPS1)). The activation of SAC transiently induces cell-cycle arrest via inhibiting the activation of APC/C. In order to establish and maintain the mitotic checkpoint, the TTK recruits many checkpoint proteins to kinetochores during mitosis via phosphorylating its substrates to ensure adequate chromosome segregation and genomic integrity [33,34]. In this way, the genomic instability from chromosome segregation defects is protected by SAC. Once the SAC is passed, the APC/C E3 ligase complex stimulates and tags cyclin B and securin for ubiquitin-mediated degradation, leading to the initiation of mitosis [5]. In a word, the checkpoints offer a failsafe mechanism to ensure the genomic integrity from the parental cell to daughter cell. The signal transduction cascade of checkpoint activation eventually converges to CDK inhibition, which indicates the CDK function as a key driver of cell-cycle progression.

### 2.2. The Roles of CDKs in Transcription

In mammals, production of messenger RNAs (mRNAs) is strictly regulated, and it is divided into discrete phases of initiation, pausing, elongation, and termination, catalyzed by RNA polymerase II (RNAPII), which is composed of a largest subunit (Rpb1) with a C-terminal domain (CTD) repeat of an evolutionarily conserved heptapeptide (Tyr–Ser–Pro–Thr–Ser–Pro–Ser) [35]. The CTD plays a vital role in RNA processing and chromatin organization in the coordination of transcriptional and co-transcriptional events through changing its phosphorylation level [36,37]. Tyr1, Ser2, Thr4, Ser5, and Ser7 are phosphorylated in the heptapeptide by multiple CDK/cyclin subunits, such as CDK1 or CDK2 and most transcriptional CDKs, such as CDK7, CDK8, and CDK9 subfamilies [38]. The phosphorylation at Ser5 and Ser7 of the CTD-RNAPII is required for the transcriptional initiation of the promoters. When the initiating transcription occurs, the Ser5 phosphorylation level decreases, while Ser2 and Tyr1 phosphorylation increases to promote transcriptional elongation. During transcription termination, Tyr1 is dephosphorylated firstly, closely followed by Ser5, Ser7, and Ser2, which permits restarting the transcription cycle [39].

**Figure 2:**
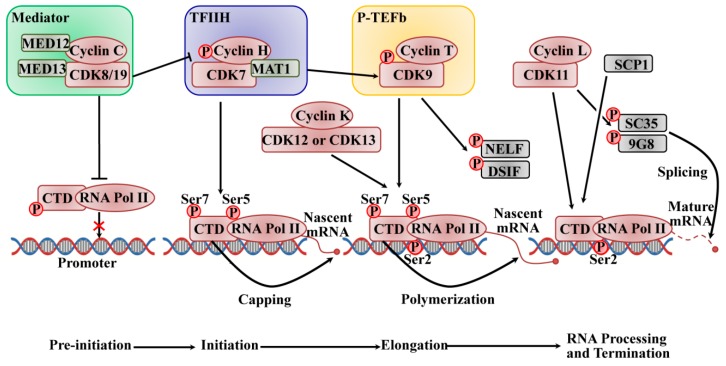
CDK/cyclin complexes regulate the RNAPII-based transcription cycle of pre-initiation, initiation, elongation, and termination. CDK8/19 activates the transcription machinery at the promoter level. CDK8/19 also phosphorylates cyclin H to inhibit the assembly of the PIC to negatively regulate the activity of TFIIH, and it phosphorylates the CTD of RNAPII to impede its binding to promoter DNA and to inhibit the assembly of the PIC. CDK7 and CDK9 drive mRNA elongation via sequential phosphorylation of the CTD-RNAPII. CDK12 and CDK13 with their cofactor cyclin K are also responsible for Ser2 phosphorylation at the CTD, allowing mRNA elongation. CDK11 is involved in the coordination between transcription and RNA splicing. DSIF and NELF inhibit elongation, while SCP1 promotes the termination of transcription. (Adapted from reference “[42], doi:10.1242/dev.091744” with permission of the journal *Development* 2013).

The active transcription is initiated by promoter recognition and DNA unwinding, thereby forming the pre-initiation complex. As shown in Figure 2, a very complicated process requires RNAPII to interact with the large multi-subunit mediator complex and several general transcription factors, and it is initiated by the binding of TATA binding protein of transcription factor II D (TFIID) to the core promoter to form the pre-initiation complex (PIC). CDK8 or CDK19 associate with C-type cyclins, which are part of the mediator complex kinase module (MED) that acts as a molecular bridge linking the gene-specific signals from DNA-bound transcription factors to the general RNAPII pre-initiation complex transcription machinery at the promoter [40,41]. The four-subunit kinase module of MED consists of CDK8 (or CDK19), cyclin C, the mediator complex subunit 12 (Med12), and Med13, and this module is commonly associated with repression of transcription. MED phosphorylates cyclin H to inhibit the assembly of pre-initiation complexes to negatively regulate the activity of transcription factor II H (TFIIH) on CTD, and it phosphorylates CTD-RNAPII to impede its binding to promoter DNA and inhibit the assembly of the PIC [42]. The TFIIH complex, which is a component of a 10-subunit general transcription factor, consists of the regulatory subunit cyclin H, the catalytic subunit CDK7, and a ring finger protein menage a trois 1 (Mat1), which functions as the helicase, ATPase, and protein kinase; it is also the last to be recruited. The DNA is unwound by its helicase activity at the transcription start site to form a single strand DNA in the RNAPII active site. The kinase activity of the CDK7 subunit in the TFIIH complex phosphorylates the Ser5 and Ser7 of CTD-RNAPII, which contributes to the initiation of transcription and promoter clearance. The phosphorylated CTD also promotes subsequent binding of a capping enzyme that catalyzes the addition of a methylguanosine cap to the 5′ end of the nascent mRNA [43]. Acting as a CDK-activating kinase (CAK), CDK7/cyclin H phosphorylates and activates CDK9, which binds to the T-type cyclins (T1 and T2) as the subunit of the positive transcription elongation factor b (P-TEFb) to release the promoter from proximal arrest and stimulate elongation. The activated CDK9/cyclin T promotes the extension of the pre-mRNA transcript through phosphorylating negative elongation factor (NELF) and 5,6-dichloro-1-beta-D-ribofuranosylbenzimidazole sensitivity-inducing factor (DSIF) to release the stalling of the elongation complex, and it phosphorylates the CTD at the serine 2 position to engage its RNA polymerizing activity [44,45]. The phosphorylation of CTD by CDK7 is required for P-TEFb recruitment, and the inhibition of CDK7 leads to a decrease in Ser2 phosphorylation by CDK9 [46]. Recent studies indicated that CDK12 and its close homolog CDK13 with their associated cofactor cyclin K are also responsible for Ser2 phosphorylation at the CTD. CDK9 phosphorylates Ser2 early on in transcription and then passes on its role to CDK12 for the majority of the elongation phase; however, the role of CDK12 in CTD phosphorylation is gene-specific [38]. Additionally, CDK12 is also involved in alternative exon splicing required for the cellular response to DNA damage, establishing a new link between the transcriptional machinery and cell-cycle regulation [47]. CDK1 phosphorylates the CTD to inhibit transcription, but the physiology needs to be addressed further [7]. CDK11/cyclin L (cycL) plays roles in transcription elongation through interacting with a variety of elongation factors, such as RNA polymerase elongation factor 2 (ELL2), general transcription factor II F (TFIIF), general transcription factor II S (TFIIS), and facilitates chromatin transcription (FACT). Additionally, CDK11/cyclin L is also involved in regulating RNA splicing through phosphorylating factors of pre-mRNA splicing, such as SC35 (Srfs2) and 9G8 (Srfs7) [42]. Sarcoplasmic calcium-binding protein 1 (SCP1) promotes transcription termination via the dephosphorylation of Ser5 of CTD-RNAPII [35]. Further studies need to explore the mechanism of dephosphorylation; however, it is reported that some CDK-counteracting phosphatases are likely to be involved, such as Cdc14 [48,49].

## 3. Dysregulation of CDKs in BC

One of the hallmarks of cancer is the uncontrolled cell proliferation via overriding many safeguards and disabling cell-cycle checkpoints associated with the dysregulation of CDK/cyclins. Until recently, a great deal of research showed the most frequent dysregulated activation alterations of the CDK/cyclins leading to the various BC phenotypes, and these are summarized in Table 1.

The CDK4/6–RB axis involved in the G1/S phase transition of the cell cycle plays an important role in BCs. Generally, cyclin D1/CDK4/6 is the key controller of RB phosphorylation to promote cell proliferation. It is expected that deregulation of the CDK4/6–cyclinD/INK4/pRB/E2F pathway or its regulators contributes toward tumorigenesis and BC maintenance [29]. Practically, the loss of INK4 and CIP/KIP family proteins, as well as the amplification of CDK4/6, was observed clinically in BC [9,14]. A recent study showed that the different BC sub-types have different cell-cycle checkpoint molecular alterations [14]. The dataset-based cancer genome study from 482 invasive BC patients showed that 27.4% of CDK4/6–RB axis genetic deregulation involves either the expression of single gene alteration or multiple gene alterations in combination [101]. In particular, estrogen can increase the rate of cell-cycle progression from the G1 to the S phase in ER-positive (ER+) BC, where the cyclin D1–CDK4/6–RB complex acts as the estrogen effector. Briefly, the estrogen binding to ER-alpha drives the transcription of cyclin D1, while the stimulation of CDK4/6 and phosphorylation of RB drive cell-cycle progression through the checkpoint, leading to initiation of the cell-cycle signal to stimulate the expression of multiple receptor-driven genes involved in cell proliferation and survival. Cyclin D1 amplification is detected in about 15% of BCs, particularly ER+ BCs [102]. Additionally, ER+ BCs often exhibit upregulated expression of estrogen receptor 1 (ESR1) protein and high expression of phosphatidylinositol-4,5-bisphosphate 3-kinase catalytic subunit alpha (PIK3CA), which contributes to cell-cycle progression through the mitogenic protein kinase B (AKT)/ mammalian target of rapamycin (mTOR) signaling pathway [14]. ER+ BC is relatively genomically stable with a primary dependency on estrogen signaling, and it typically has normal function of RB and p53 tumor suppressor pathways compared to other BC subtypes, such as HER2+ and TNBC. Similarly, HER2-induced cell growth is also mediated by the CDK4/6–RB axis [103]. The mouse models of human BC also indicate that the stimulation of the cyclin D1–CDK4/6 axis leads to a tumorigenic phenotype and contributes toward the initiation and maintenance of tumorigenesis in HER2+ BC [101]. With the highly frequent HER2+ BC, CDK4 is amplified, as well as erb-b2 receptor tyrosine kinase 2 (ERBB2, the gene encoding the HER2 receptor), mutations of tumor protein p53 (TP53), PIK3CA, and phosphatase and tensin homolog (PTEN), and cyclin D1. On the contrary, genomic, clinical, and proteomic RB pathway data in TNBC exhibit RB1 mutation or deletion in 20% of cases and cyclin E1 amplification in 9% of the cases, high-level expression of cyclin dependent kinase inhibitor 2A (CDKN2A), low expression of RB1, and high proliferation rate, as well as frequent alteration in the DNA damage response genes, such as tumor suppressor breast cancer 1 (BRCA1) [104,105]. The overexpression of cyclin E indicates a poor prognostic marker in TNBC and correlates to negative ER and PR status [106]. TNBC also activates mutations or amplification of PIK3CA, B-Raf proto-oncogene, serine/threonine kinase (BRAF), KRAS proto-oncogene, GTPase (KRAS) and/or EGFR and/or PTEN loss, resulting in the activation of an abnormal Raf/MAPK/ERK or PI3K/Akt/mTOR single pathway [107]. However, the rate of PIK3CA mutation in TNBC is only 8.3% [108]. Due to frequent loss or mutation of RB1 in TNBC, the integrity of the cell cycle is compromised, which is controlled by the Rb/E2F/CDK4/6 pathway. TNBC patients are commonly considered to be poor candidates for CDK inhibition. However, TNBC is highly sensitive to a CDK2/9 inhibitor based on a preclinical trial, indicating that there may be unknown factors involving the CDK complex in TNBC proliferation [109]. On the other hand, some studies recently indicated that the expression of multiple genes of SAC is altered in TNBC, such as TTK, BUB1, MAD2, aurora kinase B (AURKB), and DNA repair proteins, presumably due to the highly genomic instability in TNBC [5].

Additionally, other CDKs, such as CDK2, are upregulated; consequently, this often results in the amplification and/or overexpression of its partners cyclin A and cyclin E in BC [29]. CDK1 and its associated cyclins, cyclin A2 and cyclin B1, are often involved in mitotic progression, and increased expression of cyclin B1 is observed in BC [110]. However, there is no direct evidence to prove the relationship between genetic alteration dysregulating CDK1 activity and the initiation of BC. One study indicated that the loss of CDK12 protein significantly improves the phenotype of TNBC due to the loss of CDK12 resulting in defects in DNA repair [79]. The transcriptional cyclin-dependent kinase, CDK7, mediates transcriptional addition to a vital cluster of genes in TNBC, and CDK7 inhibition is a useful therapy for TNBC patients [62]. Thus, different mechanisms exist across various BC subtypes.

## 4. Targeting CDKs in BC Therapy

Given their roles in sustaining cancer cell growth, cyclins and CDKs are attractive targets for BC therapeutics. During the last decade, tremendous progress was made in developing new and effective therapies, particularly through diverting BC cells from the proliferation phenotype to the non-division state. CDK4/6 inhibitors, which mainly block the phosphorylation of RB to inhibit the cell cycle, are studied widely, and they gained the most attractive findings. CDK inhibitors are classified either as relatively non-selective pan-inhibitors or selective for one single CDK based on their specificity against CDKs. So far, CDK inhibitor drugs entered in numerous clinical trials in BC, instead of irreversible ATP-competitive (covalent) inhibition, reversible and irreversible allosteric inhibition, and antibody–drug conjugation (ADCs). These inhibitors target cell-cycle regulators in the malignant cells, providing a therapeutic window where the vulnerabilities of cancer cells may be exploited with tolerable side effects from the normal tissue toxicity.

### 4.1. The Early Pan-CDK Inhibitors in BC

Most of the early CDK inhibitors exhibit relatively nonspecific inhibition, and they are referred to as pan-CDK inhibitors. Many pan-CDK inhibitors failed before phase II trials due to their limited clinical activity as monotherapy, dose-limiting toxicities caused by undesirable target inhibition from the off-target cells, such as nausea, vomiting, fatigue, hepatic dysfunction, neuropathy, myelosuppression, and gastrointestinal effects, and the lack of predictive biomarkers of these agents for patient [111]. The early pan-CDK inhibitors include flavopiridol (also known as alvocidib, developed by Sanofi-Aventis), dinaciclib (also known as SCH 727965, developed by Merck), and seliciclib (also known as roscovitine, developed by Cyclacel), as well as mitotic kinase inhibitors such as AURKB and PLK1. It was initially indicated that CDK1, CDK2, and CDK5 were the targets of seliciclib; however, subsequent data showed that it also inhibits transcription through CDK7 and CDK9 [112].

Of these first-generation inhibitors, flavopiridol is a semi-synthetic flavonoid derived from rohitukine, a chromone alkaloid, with more than 60 clinical trials carried out between 1998 and 2014. It exerts its anticancer effect via the inhibition of CDK1, CDK2, CDK4, CDK6, CDK7, and CDK9 [113,114]. Flavopiridol causes cell-cycle arrest in G1 and G2 phase; however, later work indicated that, in certain contexts, it also induces a cytotoxic response by blocking the transcriptional activity of CDK7 and CDK9, as well as c-MYC [16]. In several phase I trials in solid malignancies, such as renal, prostate, and advanced sarcoma, flavopiridol was active as a single agent, as its combination with other chemotherapy resulted in insufficient efficacy in BC [115], whereas low levels of clinical activity were reported in phase II studies for solid tumors. However, evidence showed that flavopiridol may have clinical activity in hematological malignancies, such as chronic lymphocytic leukemia (CLL) and mantle cell lymphoma [116,117]. Seliciclib, an inhibitor of CDK1, CDK2, CDK5, and CDK7, did not show significant antitumor activities in preclinical and clinical studies as a monotherapy. Under clinical investigation in combination with chemotherapy for solid tumors, side-effects occurred commonly, such as nausea, hyperglycemia, and hypokalemia [118]. In addition, one of the most significant pan-CDK inhibitors, dinaciclib, inhibits CDK1, CDK2, CDK5, and CDK9 with superior inhibitory potency for RB phosphorylation, and it demonstrates an improved therapeutic index compared with flavopiridol [9]. In xenograft models of some solid tumors, such as ovarian and pancreatic cancers, pediatric acute lymphoblastic leukemia (ALL), and neuroblastoma-RAS viral oncogene homolog (NRAS)-mutant melanoma, it showed high activity in blocking the proliferation of tumor cells [8]. Unfortunately, the preliminary results in BC were disappointing. A phase II randomized clinical trial of dinaciclib versus capecitabine in advanced breast cancer (ABC) was stopped due to its inferior efficacy compared to capecitabine [119]. Moreover, preclinical studies suggested that the combination of dinaciclib with an anthracycline has a synergistic effect in BC cell lines, and a phase I study with dinaciclib in combination with epirubicin in metastatic BC (MBC) patients showed no responses and high toxicity [29]. Finally, dinaciclib treatment may be efficacious in MYC-overexpressing TNBC due to it inhibiting the growth of tumor and enhancing survival in preclinical mouse models [8,50]. In parallel with flavopiridol, roscovitine, a purine-based CDK inhibitor, was evaluated in the clinic. Unfortunately, only one single trial is ongoing for roscovitine in Cushing disease. Generally, first-generation pan-CDK inhibitors showed a low therapeutic index and high toxicities. Ongoing studies were developed to exploit new CDK inhibitors to circumvent these limitations.

### 4.2. The Clinical Success of CDK4/6-Selective Inhibitors in BC

The resistance induced by endocrine therapy in ER+ BC patients raised opportunities for the development of CDK inhibitors. The CDK4/6 inhibitors were effectively approved, especially when combined with anti-estrogen therapies [120], both in preclinical and clinical trials for ER+ BC. The selective CDK4/6 inhibitors, palbociclib (PD0332991), ribociclib (LEE011), and abemaciclib (LY2835219), were approved by the Food and Drug Administration (FDA) and the European Medicines Agency (EMA) for the therapy of ER+/HER2− advanced and metastatic BC (A/MBC), showing dose-dependent growth inhibition in ER+ BC. All three drugs are small-molecule, ATP-competitive drugs that can bind to the ATP-binding pocket of CDK4 and CDK6, with specific interactions with residues in the ATP-binding cleft [9]. However, abemaciclib appears to readily bind to the ATP cleft by burying two fluorine atoms against the back wall of the ATP-binding pocket, and it binds with less selectivity than ribociclib and palbociclib [121]. Palbociclib shows no activities against 36 additional kinases, whereas ribociclib demonstrates no effects against CDK1/2 for the therapy of ER+/HER2− ABC in combination with anti-estrogen therapy, such as letrozole. Palbociclib and abemaciclib have decreased potency against CDK1, CDK2, CDK7, and CDK9, and they are registered for second-line therapy with fulvestrant [122]. Reported clinical trials assessing CDK4/6 inhibitors in BC are shown in Table 2.

Palbociclib (IBRANCE; PD0332991; Pfizer; C_24_H_29_N_7_O_2_) was the first CDK4/6 inhibitor approved for BC treatment. It is a reversible, orally administered, potent, small-molecule selective inhibitor of CDK4/6 [123], with a low enzymatic half-maximal inhibitory concentration (IC_50_) of 11 nM for CDK4 and 15 nM for CDK6 [124]. In fact, the human BC cell line shows different sensitivity to palbociclib based on its phenotype. The ER+ BC cell lines with luminal features are more sensitive to palbociclib than ER− BC cells with basal-like and TNBC histology [125,126]. Palbociclib is predicted to be dependent on the presence of a functional RB protein, which causes cell-cycle arrest in G1 phase by preventing RB phosphorylation at Ser 780 via cyclin D–CDK4/6. Consistent with the notion of RB representing the major rate-limiting target of CDK4/6 in the cell cycle and the kinase selectivity profile of palbociclib, it shows no anti-proliferative activity in RB-deficient cells, where the requirement for CDK4/6 is bypassed [127]. Early trials showed palbociclib efficacy in ER+/HER2− ABC. The phase I palbociclib trial in MBC patients indicated that the preliminary, safety response and tolerable toxicity are favorable for proceeding to the next phase of studies, and myelosuppression was the main dose-limiting toxicity (DLT) [128,129]. The open-label randomized phase II clinical trial PALOMA-1 (also known as TRIO-18) studied the safety and efficacy of palbociclib (125 mg, 3/1 schedule) plus letrozole (an enzyme involved in the key step of estrogen biosynthesis) or letrozole alone for postmenopausal women with previously untreated ER+/HER2− ABC. The result showed that the median progression-free survival (PFS) for the combination of letrozole and palbociclib group was remarkably increased (from 10.2 months to 20.2 months) compared to letrozole alone, and the median overall survival (OS) was also improved with the combination treatment. Based on the results of the PALOMA-1 trial, the FDA provided accelerated (provisional) approval of palbociclib (Ibrance) in combination with letrozole for the treatment of postmenopausal women with ER+/HER2− ABC on February 3, 2015 [130]. After the promising results, the randomized, double-blind, phase III PALOMA-2 (NCT01942135) clinical trial was conducted to confirm the significant clinical benefit and safety of palbociclib plus letrozole, with a median PFS of 24.8 months in the combination therapy versus 14.5 months in the letrozole group alone. Overall survival data showed a non-significant trend toward the palbociclib group. Similarly, the mainly adverse event (AE) was neutropenia. Based on data from PALOMA-2, the FDA granted regular approval of palbociclib in combination with an aromatase inhibitor for the treatment of ER+/HER2− ABC/MBC in 2017 [131]. Finally, the randomized, double-blind, placebo-controlled phase III trial PALOMA-3 trial tested the efficacy of palbociclib with fulvestrant (ER downregulator and antagonist) by comparing treatment with palbociclib and fulvestrant to placebo and fulvestrant for patients with ER+/HER2− MBC who relapsed or progressed during prior endocrine therapy in pre- and postmenopausal stages. Palbociclib combined with fulvestrant improved the median PFS compared to fulvestrant alone, from 4.6 months to 9.5 months. Based on these results, the FDA approved palbociclib (Ibrance) for use in combination with fulvestrant for the treatment of women with ER+/HER2− ABC/MBC on February 19, 2016 [132,133,134,135]. Additionally, many ongoing studies are exploring palbociclib in ER+/HER2− early BC patients, including adjuvant and neoadjuvant studies, such as the randomized phase III study of PALLAS (NCT02513394) and PENELOPE-B (NCT01864746); primary results are expected in 2020 [6]. Furthermore, the combination of palbociclib with other targeted agents, such as tamoxifen and trastuzumab, demonstrated a synergistic interaction and efficiently suppressed the proliferation of ER+ BC cell lines [126].

Ribociclib (KISQALI; LEE011; Novartis; C_23_H_30_N_8_O) is another oral drug with high-potency bioavailable selective inhibition of CDK4/6, which lacks significant activity against CDK1 and CDK2, with a low enzymatic IC_50_ of 10 nM for CDK4 and 40 nM for CDK6 [120]. Similarly to palbociclib, it blocks RB-positive (RB+) BC cell lines through inhibiting RB phosphorylation, and it causes cell-cycle arrest of tumor cells [136]. Pharmacokinetic data were reported in a phase I ribociclib monotherapy dose escalation study (NCT01237236), showing that doses of ribociclib in monotherapy were administered from 50 to 1200 mg (3/1 schedule), as well as 600 mg (once daily, continuously) for three consecutive weeks and one week off, and the maximum tolerated dose was 900 mg from RB+ advanced solid tumors (including 18 breast cancer patients) or lymphomas, while the most common DLTs were neutropenia and thrombocytopenia [137]. In the phase II trial MONALEESA-1 (NCT01919229), postmenopausal women with ER+/HER2− early BC received letrozole with or without ribociclib (400 or 600 mg) for two weeks prior to surgery. The study showed that the ribociclib plus letrozole combination was tolerated well, and no grade 3/4 AEs were observed after treatment [138]. A randomized double-blind, placebo-controlled, phase III MONALEESA-2 (NCT01958021) trial enrolled 668 postmenopausal HR+/HER2− ABC patients who received no prior therapy for advanced disease; they received either ribociclib (600 mg/day, three weeks on/one week off) plus letrozole (2.5 mg/day) or placebo plus letrozole until the endpoints. The trial showed that the combination treatment of ribociclib and letrozole significantly prolonged PFS (from 16 months to 25.3 months) compared to letrozole alone, which led to the FDA approval of ribociclib (Kisquali) in combination with letrozole inhibitors as a first-line treatment for HR+/HER2− M/ABC on March 13, 2017 [139]. A randomized double-blind, MONALEESA-3 (NCT02422615) phase III trial was intended for postmenopausal women with HR+/HER2− ABC patients who received no or only one line of prior endocrine treatment to evaluate the efficacy of ribociclib or placebo with fulvestrant. The trial showed that the median PFS was significantly improved with the addition of ribociclib to fulvestrant compared to the placebo with fulvestrant (20.5 months versus 12.8 months). Based on this result, the FDA approved this treatment combination in HR+/HER2− M/ABC patients as first-line or second-line therapy in July 2018 [140]. Most recently, another randomized double-blind, MONALEESA-7 (NCT02278120) phase III trial was dedicated to premenopausal patients with HR+/HER2− ABC who received ovarian function suppression together with oral endocrine therapy (tamoxifen and goserelin or a non-steroidal aromatase inhibitor (NSAI) and goserelin) with or without ribociclib. The study showed that the combination of ribociclib with endocrine therapy significantly improved the median PFS with 23.8 months versus 13.0 months in the placebo with NSAI/tamoxifen plus goserelin group [141]. Additionally, there are a great deal of other ongoing trials exploring ribociclib in combination with other targeted agents for BC, such as the PACE trial (NCT03147287) and MAINTAIN trial (NCT02632045), which are being carried out to evaluate the efficacy of Ribociclib in HR+ MBC patients previously treated with a CDK4/6 inhibitor. Moreover, the TRINITI-1 trial (NCT01857193) is evaluating the effects of a triple combination of everolimus with ribociclib and exemestane-progressed patients who previously used a CDK4/6 inhibitor. The study of NCT03238196 is evaluating the effects of a triple combination of erdafitinib with ribociclib and fulvestrant in HR+ MBC [142].

Abemaciclib (VERZENIO; LY2834219; Lilly; C_27_H_32_F_2_N_8_) is an orally available bioavailable inhibitor of CDK4/6 with low enzymatic IC_50_, i.e., 2 nM for CDK4 and 10 nM for CDK6, correlating with a dose-dependent inhibition of RB phosphorylation and cell-cycle arrest at G1 phase, leading to inhibition of proliferation and decreased cell number. Abemaciclib inhibits not only CDK4/6 but also some other more complex pharmacological functions, including an effective CDK9 and Pim-1 proto-oncogene, serine/threonine kinase (PIM1), albeit with lower potency [143]. A preclinical study showed that abemaciclib can be used alone or in combination with other drugs. Abemaciclib can cross the blood–brain barrier at lower doses and may remain on target for a longer time than palbociclib. Therefore, these properties are being investigated in anti-tumor therapy for patients with brain metastases [144,145]. A phase I trial of abemaciclib monotherapy (200 mg as monotherapy or 150 mg when given with endocrine therapy) showed that the overall response rate was 31% and 61% of heavily pretreated HR+ BC patients achieving either response or stable disease lasting beyond six months, suggesting that abemaciclib may have benefit as a monotherapy [146]. The single-arm phase II study MONARCH-1 (NCT02102490) was carried to evaluate the safety and efficacy of abemaciclib alone for heavily treated HR+/HER2− M/ABC patients (brain metastases were excluded), with abemaciclib 200 mg administered on a continuous schedule every 12 h until disease progression. The study exhibited a confirmed objective response rate (ORR) of 19.7%, while the clinical benefit rate was 42.4% and median PFS was 6.0 months [147]. Based on the results, the FDA granted approval for the poor-prognosis BC patient group in September 2017. Furthermore, the randomized, double-blind, placebo-controlled MONARCH 2 (NCT02107703) phase III clinical trial was carried out for HR+/HER2− ABC patients who received prior neoadjuvant or adjuvant endocrine therapy and relapsed after endocrine therapy. Patients were randomized 2:1 and received abemaciclib or placebo with fulvestrant. The study indicated that a combination of abemaciclib with fulvestrant significant improved median PFS compared to fulvestrant alone (16.4 versus 9.3 months) [148], which led to the FDA approval of abemaciclib (Verzenio) in combination with fulvestrant in patients with HR+/HER2− M/ABC as second-line therapy in September 2017. Another phase III MONARCH 3 (NCT02246621) study treatment was proposed as a first-line treatment in HR+/HER2− MBC patients who did not receive prior treatment. The patients were randomized 2:1 to receive abemaciclib with anastrozole or letrozole compared to placebo plus anastrozole or letrozole. The results showed that the abemaciclib arm had better median PFS than the placebo arm (28.18 versus 14.76 months) [149], which also led to the FDA approving abemaciclib (Verzenio) in combination with aromatase therapy in HR+/HER2− M/ABC patients as first-line therapy in February 2018. In addition, other ongoing studies with abemaciclib in BC were carried out, such as the neoMONARCH (NCT02441946) phase II clinical trial to compare the biological effects of abemaciclib combination with anastrozole to those of abemaciclib monotherapy and anastrozole monotherapy for two weeks, as well as to evaluate the activity and safety of abemaciclib with anastrozole in a subsequent 14 weeks with HR+/HER2− ABC [150]. The monarchE study (NCT03155997) assessed the safety and efficacy of abemaciclib combined with standard endocrine therapy versus endocrine therapy alone in high-risk, node-positive, early-stage BC [131].

In addition to the FDA-approved three compounds, several other CDK4/6 inhibitors are being explored, with ongoing clinical studies evaluating their effect on their own or in combination with other targeted therapies as standard chemotherapies in BC. For instance, the international, open-label, randomized clinical trial, phase III study PEARL (NCT02028507) is being carried out to test palbociclib with endocrine therapy versus chemotherapy (capecitabine) in postmenopausal HR+/HER2− MBC, with resistance to aromatase inhibitors. The international, multicenter, randomized clinical trial, phase Ib/II study PASTOR (NCT02599714) is assessing vistusertib plus palbociclib plus fulvestrant versus placebo plus palbociclib plus fulvestrant in ER+ local A/MBC postmenopausal patients previously treated with hormonal therapy [120]. In addition to HR+/HER2− A/MBC, ongoing and recruiting clinical trials are evaluating the activity of CDK4/6 inhibitors in other BC subtypes, such as HER2+ BC and TNBC. Palbociclib/ribociclib with bicalutamide therapy was carried out for the treatment of AR+ TNBC (NCT02605486/NCT03090165). The monarcHER (NCT02675231) study also features abemaciclib under active clinical investigation for patients with HR+/HER2+ M/ABC after at least two HER2-directed therapies [131]. Some ongoing clinical trials assessing CDK4/6 inhibitors for HR+/HER2+ M/ABC or TNBC patients are listed in Table 3.

### 4.3. The Novel CDK Inhibitors in BC

With a better understanding of CDKs in different BC subtypes, as well as the achievements using CDK4/6 inhibitors in HR+/HER2− A/MBC, along with the side effects and the emergence of resistance to the present widely used inhibitors, the current exploration of new CDK inhibitors against cell-cycle targets elicits more interests.

At present, many novel CDK inhibitors were discovered. Jeong et al. found that piperlongumine (PL) inhibits ER+ BC cell proliferation and migration. PL as a natural product extracted from pepper, which inhibits the expression levels of CDK1 and CDK4/6 and induces G2/M phase cell-cycle arrest to inhibit tumorigenesis [151]. Quereda et al. found that SR-4835 is a highly selective dual inhibitor of CDK12 and CDK13, which can inhibit TNBC cell proliferation [80]. Panduratin A (PA) plays multiple roles with anti-inflammatory, antibacterial, antioxidant, and anti-cancer activity. PA also blocks the cell cycle in G0/G1 phase through dose-dependently decreasing the expression of CDK4 and cyclin D1 [152]. Vanicoside B, a phenylpropanoyl sucrose derivative of flavonoid glycoside, acted both as a PKC inhibitor and as a chemo-preventive agent in 12-*O*-tetradecanoylphorbol-13-acetate (TPA)-induced skin carcinogenesis mouse model. Kim et al. found that Vanicoside B inhibited the expression of CDK8-mediated signaling pathways and epithelial transforming proteins, and it induced cell-cycle arrest in MDA-MB-231 and HCC38 cells [153]. The protein phosphatase, Mg^2+/^Mn^2+^-dependent 1A (PPM1A), a member of Ser/Thr protein phosphatase 2C family, is involved in regulating proliferation, cell invasion, and migration through reducing CDK and RB phosphorylation in TNBC [154]. Yu et al. found that the inhibition of the subunit of the COP9 signalosome complex subunit 4 (CSN4) increases the sub-G1 cell population and induces apoptosis via regulating CDK6 in the BC cell line MDA-MB-231 [155]. β-Thujaplicin is a natural monoterpenoid that can induce G0/G1 phase cell-cycle arrest, as well as regulate cell-cycle mediators, cyclin D1, cyclin E, and CDK4, thereby inhibiting the proliferation of ER- basal-like MCF10DCIS.com human BC cells [156]. The *Fomes fomentarius* ethanol extract (FFE) arrests the S and G2/M cell populations by inhibiting the expression of cell-cycle regulatory proteins, such as CDK2, cyclin A/E, and S-phase kinase-associated protein 2 (Skp2), leading to apoptosis via targeting AKT and a reduction in the migration of MDA-MB-231 cells [157]. Recently, researchers found that a new water soluble bis(hydroxymethyl) alkanoate curcuminoid derivative, MTH-3, participates in G2/M phase arrest in MDA-MB-231 cells by downregulating the expression of CDK1 [158]. Similarly, (5, 7, 8-trihydroxyflavone (NOR-wogonin) is a polyhydroxyflavone with antitumor activity. It can significantly inhibit the proliferation of TNBC cell lines (MDA-MB-231, BT-549, HCC70, and HCC1806) compared with non-tumorigenic BC lines (MCF-10A and AG11132) through downregulating the expression of CDK1 [159]. Moreover, Galangin, another plant anticancer compound, inhibits the survival of MCF-7 cells and induces apoptosis by downregulating CDK1, CDK2, and CDK4, leading to cell-cycle arrest [160]. The 5,7-dihydroxy-2-[4′-hydroxy-3′-(methoxymethyl)phenyl]-6-C-β-glucopyranosyl flavone (from *Urginea indica* bulb) induces G0/G1 arrest and apoptosis, as well as inhibits angiogenesis in BC cells through targeting CDK1 and CDK6 [161]. Resveratrol improves the sensitivity of BC chemotherapy and prevents the development of cancer by targeting miR-122-5, and then influences the expression of CDK2, CDK4, and CDK6, resulting in cell-cycle arrest [162]. Icariin, the main component with antitumor activity extracted from *Epimedium brevicornum* Maxim, decreases the expression of CDK2 and CDK4 to cause cell-cycle arrest in tamoxifen-resistant BC cell line MCF-7/TAM [163]. Tyrosine kinase WEE1 inhibitor AZD 1775 shows strong anti-proliferative effects. Jin et al. found that the combined use of AZD 6738 and AZD 1775 activates CDK1, leading to DNA damage, mitotic defects, and cell death [164]. A microtubule-targeting agent methyl2-(-5-fluoro-2-hydroxyphenyl)-1*H*-benzo [d] imidazole-5-carboxylate (MBIC) regulates the expression of p53, and then downregulates the expression of CDK1, leading to cell death [165].

In addition, microRNAs (miRNAs) are endogenous single-stranded non-coding RNAs with a size of 20–24 nt which specifically bind to the 3’-untranslated region (3′-UTR) of the target mRNA to induce degradation of the target mRNA or inhibit its protein translation process [166]. Studies found that multiple abnormally expressed miRNAs in BC directly target CDK and participate in the regulation of tumor progression. The expression of miR-424 is reduced in most human BC specimens and cell lines, and increased expression of miR-424 reduces the expression of CDK1, thereby causing G2/M cell-cycle arrest and inhibiting cell proliferation [167]. MiR-128-3p inhibits the proliferation and motility of BC cells by affecting the expression of CDK4/6/cyclin D1 and CDK2/cyclin E1, leading to G0/G1 phase arrest [168]. MiR-122-5 also directly targets CDK2, CDK4, and CDK6, resulting in cell-cycle arrest [162]. MiR-141-3p is downregulated in the trastuzumab-resistant cell line and deregulated expression of miR-141-3p/CDK8 reduces drug resistance and inhibits cell migration and invasion [169]. Liu et al. found that the upregulation of hsa_circ_0136666 promotes the progression of BC by spongifying miR-1299 and targeting CDK6 to inhibit the proliferation, migration, and invasion of BC [170]. Moreover, Zheng et al. found that long non-coding RNA CASC2/miR-18a-5p/CDK19 is involved in BC chemical resistance [100] and the non-coding RNA 00511 (LINC00511)/miR-29c/CDK6 is involved in paclitaxel cytotoxicity in BC cells [171]. Taken together, these miRNAs can be used as candidate targets for novel CDK inhibitors in BC therapeutic purposes with miRNA antagonists (also called antagomirs or antimiRs, gene-silencing therapy) or miRNA mimics (also known as miRNA replacement therapy, replacement therapy).

### 4.4. The Combined Treatment with CDK Inhibitors and Other Agents

BC is a challenging solid cancer type, and the monotherapy CDK inhibitors therapy may lead to many defects; thus, CDK inhibitors combined with other clinical agents may gain a synergistic treatment and good outcome. Recently, the sequential treatment of palbociclib/paclitaxel inhibited cell proliferation and increased cell death more efficaciously than single treatments. Paclitaxel inhibits palbociclib-mediated AKT induction and downregulates the RB/E2F/c-myc signaling pathway. The sequential combination of palbociclib/paclitaxel can enhance the inhibitory effects on glucose metabolism, and pretreatment with palbociclib can significantly improve the therapeutic effect of chemotherapy [172]. However, the simultaneous use of palbociclib and paclitaxel produces an antagonistic effect. Kettner et al. found that targeting interleukin 6 (IL6)/signal transducer and activator of transcription 3 (STAT3) and DNA repair deficiency using a combination STAT3 and poly(ADP-ribose) polymerase (PARP) inhibitor could effectively treat palbociclib-resistant ER+ BC [173]. Messer et al. reported a case of the combined use of palbociclib and radiotherapy to enhance the effect of radiotherapy, with improved survival and reduced cell proliferation by G1 cell-cycle arrest [174]. In MDA-MB-231 cells, PTC-209 and palbociclib exhibited more profound dose-dependent cytotoxic effects, leading to inhibition of insulin signaling, focal adhesion, DNA damage response, and Wnt/pluripotency signaling, thereby reducing colony and sphere formation, cell migration, and cell viability [175]. Furthermore, the PI3K/AKT/mTOR signal pathway is an important pathway in ER+ BC. Thus, combining a CDK4/6 inhibitor with an aromatase or ribociclib and PI3K inhibitor alpelisib (BYL719) brought about enhanced tumor regression and improved the PFS versus single-agent treatment [176]. However, recent data showed that the application of PI3K inhibitors seems unsatisfactory due to its modest effects and great toxicities, while everolimus, an mTOR inhibitor, evidently improves PFS when added to endocrine therapy (ET) with less toxicity [177]. On the other hand, recent data indicated that the CDK-RB-E2F pathway was reactivated in CDK4/6 inhibitor-resistant BC cell lines, but it was sensitive to mammalian target of rapamycin complex1/2 (mTORC1/2) inhibitors. Hence, the combined use of mTORC1/2 inhibitors and a CDK4/6 inhibitor will be more effective in terms of E2F-dependent transcription and cell proliferation inhibition to overcome the resistance to CDK4/6 inhibitors [178].

## 5. Conclusions

It is abundantly clear that CDK complexes have central roles in cell proliferation, gene transcription, and cell-cycle progression control, forming a system to regulate the cell-cycle-promoting activity in response to various intracellular scenarios and extracellular signals. Continued research into the role of cell-cycle dysregulation in BC led to the identification of their potential as attractive targets for cancer therapy. Many novel CDK inhibitors enabled cell-cycle studies to be brought from bench to bedside. From initial unsatisfactory results in clinical trials with non-selective CDK inhibitors to successful selectively specific inhibitors, the treatment landscape of ER+/HER2− M/ABC was developed fundamentally over the last few decades. Novel treatment modalities that target multiple components in the same signal pathway, such as the miRNA antagonists or miRNA mimics, may help us to achieve more sustained therapeutic benefit. Indeed, the miR-34 mimic, MRX34, which targets the transcripts of multiple cell-cycle genes, entered clinical phase I evaluation recently [8]. However, resistance and the increased cost require the development of more therapeutic strategies and rational designs. The combination of CDK4/6 inhibitors with other compounds in adjuvant therapies for different sub-types of BC deserves more attention.

## Authors Contributions

Conceptualization and design (L.D., J.C., W.L., Q.C.); collection and/or assembly of data (L.D., J.C., W.L., H.C., X.X., H.A., Q.C.); manuscript writing (L.D., J.C., W.L., Q.C.); final approval of manuscript (all authors).

## Figures and Tables

**Table 1:** Summary of the biological functions of CDKs in BCs.

CDKs	Partners	Established Functions	Biological Functions in BCs	Reference
CDK1	Cyclin A/B	Associates with M phase of cell cycle	Associates with apoptosis of MYC-driven TNBC	[50,51,52]
CDK2	Cyclin A/E	Associates with G1/S phase of cell cycle	Correlates with BC or TNBC phenotype	[52,53,54]
CDK3	Cyclin C	Associates with G0/G1 and G1/S cell cycle transitions	Associates with BC cell migration, invasion, proliferation, and apoptosis	[27,55,56]
CDK4/6	Cyclin D	Associates with the G1/S phase transition of the cell cycle	Contributes toward BC initiation and maintenance of tumorigenesis	[19,29]
CDK5	p35 and p39	Drives progression from G1/S and in RB phosphorylation; linked to chemotherapy resistance and immune response	Associates with ROS-mediated cell death in BC; essential for TGF-β1-induced epithelial–mesenchymal transition	[57,58,59,60,61]
CDK7	Cyclin H	Associates with CAK and RNAPII transcription	Mediates transcriptional addiction to a vital cluster of genes in TNBC	[62,63]
CDK8	Cyclin C	RNAPII transcription in complex; regulates the initiation of transcription	Responds to adjuvant therapy in BC; associated with tumor progression	[64,65,66,67]
CDK9	Cyclin T	RNAPII transcription; promotes elongation of transcription	A prognostic biomarker in patients with BC following neoadjuvant chemotherapy	[45,68,69]
CDK10	Cyclin M	Regulates ETS2 transcription, but not through RNAPII phosphorylation	Correlates with lymph node metastasis; resistance to endocrine therapy	[70,71,72]
CDK11	Cyclin L	Regulates RNA transcription and splicing, autophagy, and apoptosis	Associates with growth and angiogenesis, proliferation, and apoptosis	[73,74,75,76,77]
CDK12	Cyclin K	Controls alternative last exon splicing; regulates the expression of DNA damage, stress, and heat shock genes	Promotes BC cell invasion, an important therapeutic implication for TNBC; drives BC initiation and trastuzumab resistance	[47,78,79,80]
CDK13	Cyclin K	Transcript synthesis toward the middle and 3′ end of the emerging RNA	Associated with DNA damage repair, genomic instability	[47,81,82]
CDK14	Cyclin Y	Promotes Wnt/β-catenin signaling through phosphorylation of the LRP6 co-receptor	Associated with cell proliferation and invasion	[83,84,85,86]
CDK15	Cyclin Y	Participates in hepatitis B virus-driven transformation	Associated with BC cell invasion and metastasis	[87,88]
CDK16	Cyclin Y	Regulates mitosis, apoptosis, and growth; synaptic trafficking and remodeling	Associated with TRAIL	[89,90,91,92]
CDK17	Cyclin Y	Promotes amyloid precursor protein-dependent Alzheimer; inhibits autophagy	Genetic expression profiles and chromosomal alterations	[93,94,95]
CDK18	Cyclin Y	Promotes amyloid precursor protein-dependent Alzheimer; inhibits autophagy; promotes DNA replication stress and stability	Increases sensitivity to replication stress-inducing chemotherapeutic agents; induces DNA replication stress	[93,94,96,97,98]
CDK19	Cyclin C	CDK8 paralog, with a similar role to CDK8, but seems to perform some distinct roles	The chemoresistance of BC; provides potential targets for the improving chemotherapy	[99,100]
CDK20	Cyclin H	Activates ICK or β-catenin–TCF signaling to stimulate cell-cycle progression	The role of CDK20 needs to be further addressed in BC	[7,35]

Abbreviations: MYC—MYC proto-oncogene, bHLH transcription factor; ROS—reactive oxygen species; TGF-β1—transforming growth factor beta 1; ETS2—ETS proto-oncogene 2, transcription factor; Wnt—wingless/integrated; LRP6—LDL receptor related protein 6; TRAIL—tumor necrosis factor (TNF)- related apoptosis-inducing ligand; ICK—intestinal cell kinase; TCF—T cell factor.

**Table 2:** Reported clinical trials assessing CDK4/6 inhibitors in BC.

Trial Name	Phase	Treatment Arms	Population	No.	PFS	Most Common AEs
PALOMA-1/ TRIO-18 (NCT00721409)	II	1. Palbociclib + letrozole 2. Letrozole	Postmenopausal women, first-line treatment for ER+/HER2−ABC	177	1. 20.2 months 2. 10.2 months	Neutropenia, leukopenia, fatigue anemia, nausea
PALOMA-2 (NCT01740427)	III	1. Palbociclib + letrozole 2. Placebo + letrozole	ER+/HER2− ABC	666	1. 19.3 months 2. 12.9 months	Neutropenia, leukopenia, nausea, fatigue, arthralgia, alopecia
PALOMA-3 (NCT01942135)	III	1. Palbociclib + fulvestrant 2. Placebo + fulvestrant	HR+/HER2− ABC	521	1. 9.2 months 2. 3.8 months	Neutropenia, leukopenia, thrombocytopenia, fatigue, nausea, headache, alopecia
PALLAS (NCT02513394)	III	1. Palbociclib for 2 years + standard adjuvant ET for 5 years 2. Standard adjuvant ET for 5 years	ER+/HER2− early BC	5795	No detailed data	No detailed data
PENELOPE-B (NCT01864746)	III	1. Palbociclib, 125 mg once daily, 28-day cycle for 13 cycles 2. Placebo 28-day cycle for 13 cycles	HR +/HER2− normal primary BC with high relapse risk after neoadjuvant chemotherapy	1250	No detailed data	No detailed data
MONALEESA-1 (NCT01919229)	II	1. Letrozole + ribociclib. 2. Letrozole	Postmenopausal women with HR+/HER2− early BC	14	Mean decrease in Ki67-expressing cells, 1. 92%, 2. 69%	Nausea, decreased appetite, diarrhea, abdominal pain, fatigue, asthenia
MONALEESA-2 (NCT01958021)	III	1. Ribociclib + letrozole 2. Placebo + letrozole	Postmenopausal women with HR+/HER2− MBC received no prior therapy for advanced disease	668	1. 19.3 months 2. 14.7 months	Neutropenia, leukopenia, nausea, fatigue, diarrhea, alopecia
MONALEESA-3 (NCT02422615)	III	1. Ribociclib + fulvestrant 2. Placebo + fulvestrant	Postmenopausal women with HR+/HER2− ABC received no or only one line prior endocrine treatment	726	1. 20.5 months 2. 12.8 months	Neutropenia, leukopenia, nausea, fatigue, diarrhea, alopecia, vomiting, constipation, arthralgia, cough, headache, rash, anemia
MONALEESA-7 (NCT02278120)	III	1. Ribociclib + NSAI/tamoxifen + goserelin 2. placebo + NSAI/tamoxifen + goserelin	Premenopausal or perimenopausal women with ER+/HER2−ABC	672	1. 23.8 months 2. 13.0 months	Neutropenia, leukopenia, increased ALT, increased AST, anemia, hypertension
MONARCH-1 (NCT02102490)	II	Abemaciclib	heavily treated HR+/HER2− M/ABC patients (brain metastases were excluded)	132	6.0 months (95% confidence interval (CI) 4.2 to 7.5)	Leucopenia, neutropenia, diarrhea, fatigue, nausea, hypokalemia, increased ALT, decreased appetite, hyponatremia, abdominal pain, thrombocytopenia
MONARCH-2 (NCT02107703)	III	1. Abemaciclib + fulvestrant 2. Placebo + fulvestrant	HR+/HER2− locally advanced or metastatic BC.	669	1. 16.4 months 2. 9.3 months	Neutropenia, diarrhea, nausea, fatigue, abdominal pain
MONARCH-3 (NCT02246621)	III	1. Abemaciclib + anastrozole/ letrozole 2. Placebo + anastrozole/ letrozole	Postmenopausal women HR+/HER2− locoregionally, recurrent, or MBC	493	1. 28.2 months 2. 14.8 months	Neutropenia, diarrhea, nausea, fatigue, infections

Abbreviations: ER—estrogen receptor; HER2—human epidermal growth factor 2; ABC—advanced breast cancer; MBC—metastatic breast cancer; No.—number; ET—endocrine therapy; PFS—progression-free survival; AEs—adverse events; NSAI—nonsteroidal aromatase inhibitor; ALT—alanine aminotransferase; AST—aspartate aminotransferase.

**Table 3:** Ongoing clinical trials assessing CDK4/6 inhibitors for HR+/HER2+ M/ABC or TNBC patients.

Trial Name	Phase	Status	Design	Treatment Arms	Population	Pts Enrolled	Objectives
NCT02947685	III	R	Randomized, parallel assignment, open label	1. Palbociclib + anti-HER2 therapy (trastuzumab/pertuzumab) + ET (letrozole, anastrozole, exemestane, fulvestrant) 2. Anti-HER2 therapy (trastuzumab/pertuzumab) + ET (letrozole, anastrozole, exemestane, fulvestrant)	HER2+/ER+ BC	496 (estimated)	PFS, OS, ORR, DOR, CBR, safety, 3 and 5 year survival probabilities
NCT02774681	II	Terminated	Single group assignment, open label	1. palbociclib PO 2. palbociclib PO + trastuzumab IV	HER2+/PR− MBC with brain metastasis	12 (estimated)	AEs, CNS, PFS, OS, CNS, ORR, safety, tolerability
NCT02530424	II	Active, N/R	Single group assignment, open label	(Trastuzumab + Pertuzumab + Palbociclib ± Fulvestrant) + Surgery	ER+/HER2+ BC suitable for neoadjuvant therapy	102 (actual)	PCR, COR, safety, tolerability
NCT02657343	Ib/II	Active, N/R	Non- randomized, parallel assignment, open label	1. Ribociclib + T-DM1 2. Ribociclib + Trastuzumab 3. Ribociclib + Trastuzumab + Fulvestrant	HER+ A/MBC	26 (actual)	Mtd, RP2D,CBR, ORR, PFS, OS.
NCT03913234	I/II	Not yet R	Single group assignment, open label	Ribociclib + Trastuzumab + Letrozole	Postmenopausal HER2+ MBC	95 (estimated)	PFS,OS, RT, QOL
NCT03054363	Ib/II	R	Non- randomized, single group assignment, open label	Tucatinib + Palbociclib + Letrozole	HR+/HER2+ A/MBC	25 (estimated)	AEs, PFS
NCT03993964	II	Not yet R	Single group assignment, open label	Pyrotinib + SHR6390	HER2+ ABC	20 (estimated)	ORR, PFS, OS
NCT03090165	I/II	Active,N/R	Single group assignment, open label	1. bicalutamide + ribociclib 400mg PO daily on days 1-21 of a 28-day cycle. 2. bicalutamide + ribociclib 400mg PO daily on days 1-28 of a 28-day cycle. 3. bicalutamide + ribociclib 600mg PO daily on days 1-21 of a 28-day cycle.	AR+ TNBC	11 (actual)	ORR, DOR, safety, tolerability, PFS, OS, CBR,
NCT03805399	Ib/II	R	Non- randomized, open label, umbrella study, parallel assignment	1. Pyrotinib + Capecitabine 2. AR inhibitor + CDK4/6 inhibitor 3. anti PD-1 + nab-paclitaxel 4. PARP inhibitor 5. BLIS + anti-VEGFR 6. MES + anti-VEGFR 7. mTOR inhibitor + nab-paclitaxel	TNBC	140 (estimated)	ORR, DOR, PFS, OS
NCT03519178	II	R	Non- randomized, single group assignment, open label	1. PF-06873600 2. PF-06873600 + Endocrine Therapy 1 3. PF-06873600 + Endocrine Therapy 2	HR+/HER2− MBC, TNBC	220 (estimated)	DL, safety, tolerability, ORR, Cmax, Tmax, PK
NCT02907918	II	R	Single group assignment, open label	(Palbociclib + letrozole + trastuzumab +/- goserelin) + surgery	ER+/HER2+ Stage II-III BC	48 (estimated)	PCR, safety, tolerability
NCT02605486	I/II	R	Single group assignment, non- randomized, open label	Palbociclib + Bicalutamide	AR+/ER− MBC	51 (estimated)	RP2D, PFS, ORR, CBR, safety, tolerability
NCT02675231	II	Active, N/R	Randomized, parallel assignment, open label	1. Abemaciclib + Trastuzumab + Fulvestrant 2. Abemaciclib + Trastuzumab 3. Trastuzumab + Standard of Care Chemotherapy	HR+/HER2+ A/MBC	225 (estimated)	PFS, OS, CR, PR, DOR

Abbreviations: Pts—Patients; ABC—advanced breast cancer; MBC—metastatic breast cancer; TNBC—triple-negative breast cancer; R—recruiting; N/R—not recruiting; ET—endocrine therapy; PO—peros; T-DM1—trastuzumab emtansine/Kadcyla; PD-1—programmed cell death protein 1; PARP—poly(ADP-ribose) polymerase; BLIS—basal-like immune suppressed; VEGFR—vascular endothelial growth factor receptor; MES—mesenchymal; mTOR—mammalian target of rapamycin; AR—androgen receptor; PFS—progression-free survival; OS—overall survival; ORR—overall response rate; DOR—duration of response; CBR—clinical benefit rate; CNS—central nervous system; PCR—pathological complete response; Mtd—maximum tolerated dose; RP2D—recommended phase 2 dose; AEs—adverse events; Cmax—maximal concentration; Tmax—time to maximum plasma concentration; PK—pharmacokinetic; CR—complete response; PR—partial response; COR—clinical objective response; RT—response rate; QOL—quality of life; DL—dose limit.
